# OmicsQ: a user-friendly platform for interactive quantitative omics data analysis

**DOI:** 10.1093/bioinformatics/btaf660

**Published:** 2025-12-17

**Authors:** Xuan-Tung Trinh, André Abrantes da Costa, David Bouyssié, Adelina Rogowska-Wrzesinska, Veit Schwämmle

**Affiliations:** Department of Biochemistry and Molecular Biology, University of Southern Denmark, 5230 Odense, Denmark; Department of Biochemistry and Molecular Biology, University of Southern Denmark, 5230 Odense, Denmark; BioISI – Instituto de Biosistemas e Ciências Integrativas, Faculdade de Ciências da Universidade de Lisboa, 1749-016 Lisbon, Portugal; Infrastructure Nationale de Protéomique, ProFI, UAR 2048 Toulouse, France; Institut de Pharmacologie et de Biologie Structurale (IPBS), CNRS, Université de Toulouse (UT), 31077 Toulouse, France; Department of Biochemistry and Molecular Biology, University of Southern Denmark, 5230 Odense, Denmark; Department of Biochemistry and Molecular Biology, University of Southern Denmark, 5230 Odense, Denmark

## Abstract

**Motivation:**

High-throughput omics technologies generate complex datasets with thousands of features that are quantified across multiple experimental conditions, but often suffer from incomplete measurements, missing values, and individually fluctuating variances. This requires analytical tools for accurate, deep and insightful biological interpretation, capable of dealing with a large variety of data properties and different amounts of completeness. Software capable of handling such data complexity and integrating with external applications for downstream analysis remains rare and mostly relies on programming-based environments, limiting accessibility for researchers without computational expertise.

**Results:**

We present OmicsQ, an interactive, web-based platform designed to streamline quantitative omics data analysis. OmicsQ provides an intuitive, browser-based visualization interface that integrates established statistical processing tools. Those include robust batch correction, automated experimental design annotation, and handling of missing data without imputation, which maintains data integrity and avoids artifacts from *a priori* assumptions. OmicsQ seamlessly interacts with external applications (e.g. PolySTest, VSClust, ComplexBrowser) for statistical testing, clustering, analysis of protein complex behavior, and pathway enrichment, offering a comprehensive and flexible workflow from data import to biological interpretation that is broadly applicable across domains.

**Availability and implementation:**

OmicsQ is implemented in R and Shiny and is available at https://computproteomics.bmb.sdu.dk/app_direct/OmicsQ. Source code and installation instructions: https://github.com/computproteomics/OmicsQ, DOI: 10.5281/zenodo.17778420.

## Graphical abstract

**Figure:**
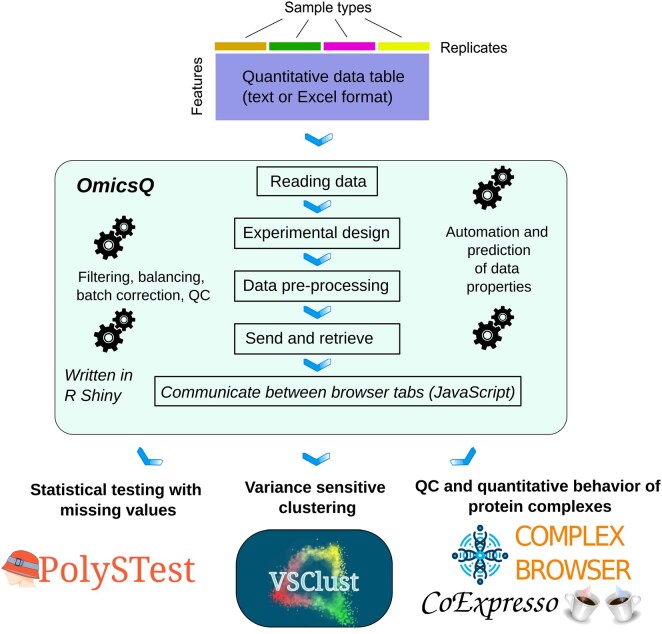

## 1 Introduction

The rapid advancements in high-throughput omics technologies have generated an unprecedented volume of quantitative data, necessitating robust and scalable computational solutions for meaningful interpretation (Aebersold and Mann 2016). However, challenges such as missing values, batch effects, and proper data normalization complicate downstream analyses and can introduce biases in the biological interpretation if not handled correctly (Karpievitch *et al.* 2012).

Missing values frequently distort statistical inference in omics studies. Naïve imputation can impose artificial structure and reduce variance, thereby inflating false discovery rates (Karpievitch *et al.* 2012, Lazar *et al.* 2016). Analyses that operate directly on incomplete matrices avoid these biases, yet user-friendly, imputation-free tools remain scarce. Batch effects, systematic differences between acquisition runs, can mask genuine biological signals if left uncorrected (Leek *et al.* 2010). Widely used batch correction methods such as ComBat (Johnson *et al.* 2007) and limma’s removeBatchEffect (Ritchie *et al.* 2015) are effective, but they may over-adjust when batch and biology are confounded. Both methods extend beyond their microarray origins to general quantitative data. Normalization counters non-biological biases. Whether using global scaling, LOWESS regression or ANOVA-based models, one must strike a balance: to remove unwanted variation without suppressing true signal (Karpievitch *et al.* 2012). The detection of batch effects (Akulenko *et al.* 2016) and interactive visualization can show the necessity and impact of different normalization and batch correction methods and thus help with their careful application.

Given the myriad of tools for quantitative analysis, and particularly their availability as libraries of scripting languages like R and Python, end users without programming skills often rely on simplified solutions. Such software frequently omits recent and more powerful methods for data treatment, creating accessibility barriers for experimental researchers, and providing only a single, rigid workflow. Moreover, instead of relying on a one-workflow-fits-all solution, end users often prefer to interact with the data to test different hypotheses and to confirm *a priori* knowledge about the given biological system.

Similar approaches like Analyst Suites (Shah *et al.* 2020, Zhang *et al.* 2023, Hsiao *et al.* 2024) target downstream analysis of proteomics data but are tied to specific upstream pipelines. DAPAR/ProStaR (Wieczorek *et al.* 2017) provide a Shiny GUI around a rich proteomics toolkit including normalization, imputation, and differential testing, but analyses typically proceed on imputed data. Perseus (Tyanova *et al.* 2016) offers a broad desktop environment for proteomics with normalization, pattern recognition, imputation and time-series modules. mixOmics (Rohart *et al.* 2017) focuses on multivariate multi-omics integration and feature selection within R, rather than end-to-end QC and preprocessing in a GUI. MetaboAnalyst (Pang *et al.* 2024) is a mature web platform primarily for metabolomics with extensive normalization and imputation options. We believe that current tools lack modularity and the ability to extensively interrogate the data using modern statistical approaches including variance-sensitive clustering and analysis at the protein complex level.

OmicsQ is designed to address these challenges by offering an intuitive, interactive, browser-based platform that streamlines key data-processing steps and exports results to external applications for downstream analyses such as statistical testing, variance-sensitive clustering, and protein quantification. By integrating automated experimental design annotation, direct handling of missing values, and interactive quality control visualization, OmicsQ empowers researchers to focus on biological interpretation rather than overcoming computational hurdles. Additionally, OmicsQ seamlessly integrates with external applications for imputation-free statistical testing, clustering, and protein complex analysis, enabling a comprehensive workflow from data import to functional insights. This includes clustering applicable to incomplete data and statistical testing that includes a presence-absence model. By bridging the gap between computational complexity and usability, OmicsQ provides an accessible and reliable solution for quantitative omics data analysis.

## 2 Technical methods

**Figure 1.**
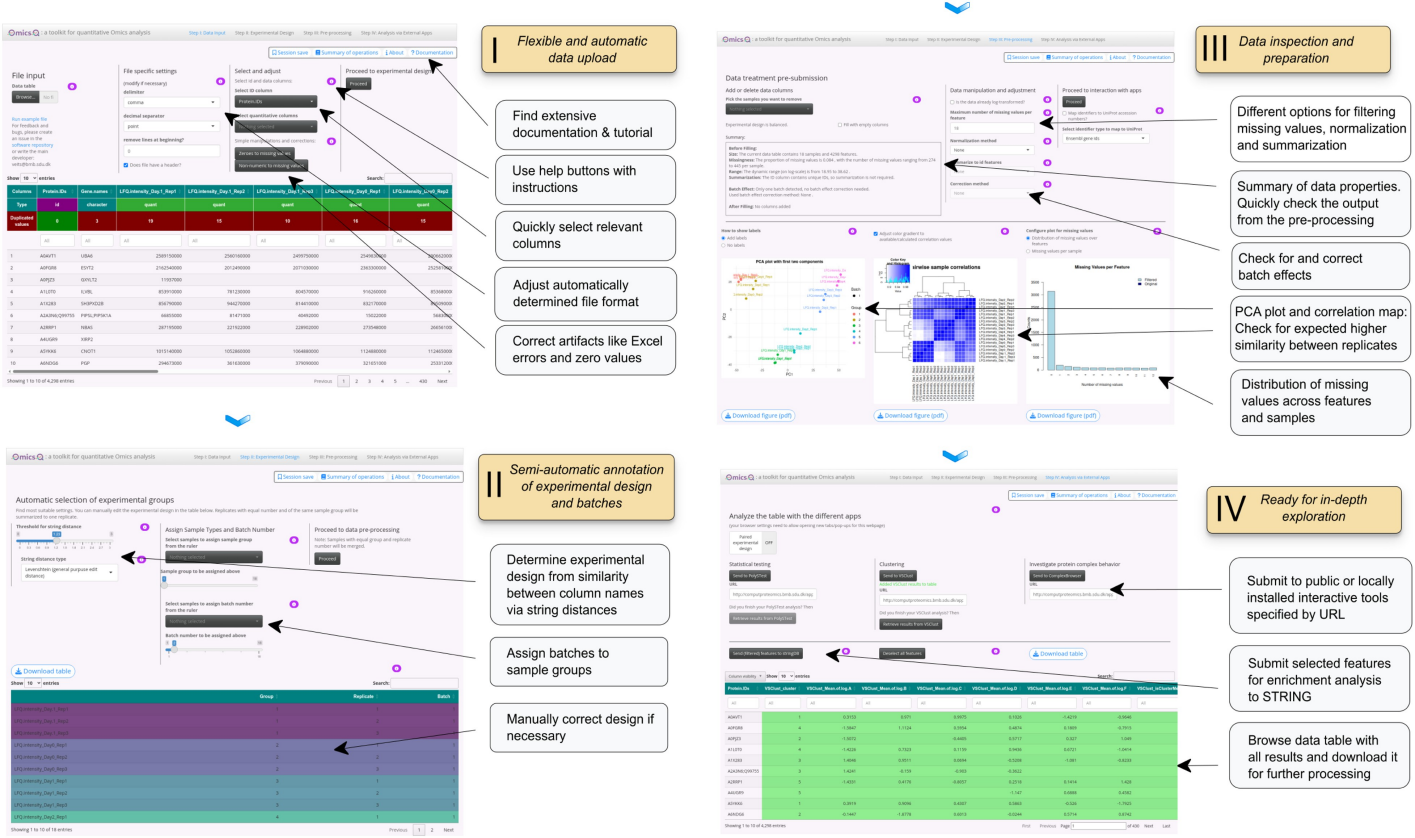
Overview of OmicsQ features. Diagram illustrating the key steps in the OmicsQ workflow: data upload, experimental design annotation, pre-processing (including batch correction and missing value handling), and quality control, and interaction with PolySTest, VSClust and ComplexBrowser.

OmicsQ was implemented as an interactive, user-friendly interface built with the Shiny framework, featuring real-time feedback through data summaries, projection onto principal components and correlation plots. This allows users to directly observe the impact of data-processing choices, and thus should enable informed decisions before continuing with in-depth analysis such as statistical testing and variance-sensitive clustering (Fig. 1).

By using an interactive interface with automatic data property detection and extensive help features, OmicsQ aims to provide clear guidance through critical analysis steps, beginning with file import. Upon uploading Excel or CSV/TSV tables containing features quantified across different samples, such as experimental conditions and replicates, OmicsQ automatically detects file formats, and permits manual adjustments when necessary. Users then assign specific columns of the uploaded data matrix as identifiers or quantitative measurements for downstream analysis.

Annotation of experimental sample groups and replicates can be a cumbersome task when dealing with many samples. OmicsQ streamlines annotation through semi-automatic comparison of sample names using multiple string-distance metrics (e.g. Levenshtein, Jaro-Winkler). This automatically creates separate groups of samples with similar names from the file header. Users can interactively adjust thresholds to optimize grouping accuracy rapidly, significantly simplifying experimental design annotation. Samples with consistent naming are easily recognized and grouped with minimal manual effort, reducing the need for time-consuming and error-prone manual annotations across tens or even hundreds of samples.

Advanced pre-processing options include direct handling of missing values without requiring imputation, which would add potentially incorrect values to the dataset. Batch effects can be detected by calcBatchEffects method from the BEclear package (Akulenko *et al.* 2016). Users can then choose whether to perform batch correction in OmicsQ with two commonly used methods from the ComBat and limma R packages (Ritchie *et al.* 2015), and visually evaluate using an integrated principal component analysis (PCA) plot and a correlation heatmap that can be downloaded as pdf files. Furthermore, there are different options for filtering, summarization of main features and normalization. OmicsQ provides a summary of the dataset and its main features such as balancing, potential batch effects and dynamic range.

## 3 Features and external tools

A significant feature of OmicsQ is its integration with external specialized statistical analysis applications. These tools are specifically tailored to address the challenges posed by high variability and limited statistical power in omics data, being able to run without requiring data completeness or relying on imputation methods based on stringent assumptions.

To facilitate seamless interaction among these tools, OmicsQ uses JavaScript messaging with these applications. The default settings call the publicly available instances on https://computproteomics.bmb.sdu.dk. Alternatively, one can direct the in-depth analysis to local installations. Docker-based containerization of all five Shiny apps ensures straightforward local deployment.

The following methods and their Shiny applications are integrated with OmicsQ:

*PolySTest* (Schwämmle *et al.* 2020) performs multi-group comparisons on incomplete matrices. Its Miss Test uses missingness patterns to detect differentially regulated features without imputation, allowing statistically rigorous hypothesis testing across standard experimental designs. PolySTest is also available as a Bioconductor R package.

*VSClust* (Schwämmle and Jensen 2018) is a variance-sensitive clustering algorithm that identifies co-regulated features even in noisy, partially missing datasets. Distances are computed against complete cluster centres, and integrated variance estimation improves sensitivity to subtle expression trends.

*ComplexBrowser* (Michalak *et al.* 2019) quantifies known protein complexes from subunit co-abundance. OmicsQ auto-converts gene or protein identifiers via the UniProt API (UniProt Consortium 2025), extending the method beyond proteomics to transcriptomics and other genomics-based data types.

*CoExpresso* (Chalabi *et al.* 2019) (invoked from ComplexBrowser for human datasets) benchmarks observed co-regulation against >200 ProteomicsDB (Lautenbacher *et al.* 2022) tissues and >3000 cancer samples in PDC (Thangudu *et al.* 2024). The recent extension to include PDC allows more extensive complex validation and new hypothesis generation.

With the integration of above applications, users can seamlessly transfer pre-processed data directly to PolySTest for statistical testing, VSClust for variance-based clustering, and ComplexBrowser for protein-complex analysis. When dealing with human data, the general behavior of protein complexes can further be investigated in CoExpresso. Results from statistical testing and clustering can be retrieved and integrated back into OmicsQ, consolidating analyses within a unified environment. Furthermore, OmicsQ uses the String API (Szklarczyk *et al.* 2019) to run functional enrichment and visualize the respective protein–protein interaction networks.

With its interactivity and modularity, OmicsQ supports flexible, interactive and user-friendly quantitative analysis applicable to diverse quantitative omics studies, such as proteomics, transcriptomics, metabolomics, and associated data types. Moreover, the software features a tutorial including a representative workflow exemplifying the entire analysis.

## 4 Discussion and conclusion

OmicsQ offers an interactive and accessible platform that can be operated entirely in a web browser. This lowers the barrier for experimental researchers, clinicians, and students who may lack coding experience but still need to perform high-quality, quantitative omics analyses with confidence. For instance, a biologist analyzing proteomics data from a small cohort of patient samples can quickly upload their dataset, correct batch effects, inspect data variability, and proceed to pathway enrichment, without needing to write a single line of code. Unlike many other tools, OmicsQ does not rely on imputation, and thus preserves data integrity for more reliable downstream analyses, as it does not add potentially incorrect information and lead to misleading results from a significance analysis.

The platform is designed to promote exploratory data analysis, enabling users to iteratively test alternative hypotheses and make informed decisions throughout their workflow. The integration with multiple external applications allows for flexible extension into statistical testing, clustering, and protein complex analysis and will be maintained to upkeep with respective updates. Integration with additional external applications and community standards, e.g. for sample metadata, will be considered in the future.

A key strength of OmicsQ is its capacity to maintain a balance between automation and interactivity. Users can inspect each transformation step, review visual summaries (e.g. PCA, heatmaps), and trace parameter choices for enhanced reproducibility. The summary of processing steps ensures that users are aware of every operation performed during the analysis.

The interactive and modular setup of OmicsQ also allows integrated analysis of different omics types when the same samples are used for data acquisition. The robustness of combinatorial statistical testing in PolySTest and variance controlled clustering in VSClust allows analysis after aggregation of the results in the same data table, and so enables identification of common biological processes. Given the often very different nature of different omics technologies and difficulties in aligning their features, such applications will need to be executed carefully.

Despite its broad applicability, OmicsQ has some limitations. Scalability remains a challenge when dealing with very large datasets, both in terms of memory usage and response time of the interface. The current implementation is optimized for medium-sized datasets (tens to hundreds of samples), typical of many proteomics and transcriptomics studies. OmicsQ and its associated applications can process datasets with thousands of features at low computational cost and can analyze data with up to 100k features in under an hour on the server (AMD EPYC 9224 24-Core Processor, 256GB RAM). Work is ongoing to introduce more efficient data handling and scalable processing beyond simple multi-threading to improve performance.

In summary, OmicsQ provides a robust, user-friendly environment for analyzing quantitative omics data. It empowers researchers to carry out essential preprocessing, hypothesis testing, and functional interpretation tasks without requiring programming skills. By bridging usability with analytical rigor, OmicsQ contributes to more reproducible and insightful omics research workflows.
